# The role of MRI in cervical cancer > 2 cm (FIGO stage IB2-IIA1) conservatively treated with neoadjuvant chemotherapy followed by conization: a pilot study

**DOI:** 10.1007/s11547-021-01377-1

**Published:** 2021-05-31

**Authors:** Luca Russo, Benedetta Gui, Maura Miccò, Camilla Panico, Rosa De Vincenzo, Francesco Fanfani, Giovanni Scambia, Riccardo Manfredi

**Affiliations:** 1 UOC Radiologia Generale ed Interventistica generale, Area Diagnostica per Immagini, Dipartimento Diagnostica per Immagini, Radioterapia Oncologica ed Ematologia, Fondazione Policlinico Universitario A. Gemelli IRCCS, Rome, Italy; 2 UOC Ginecologia Oncologica, Dipartimento per la Salute della Donna, del Bambino e di Sanità Pubblica, Fondazione Policlinico Universitario A. Gemelli IRCCS, Rome, Italy; 3 Dipartimento Universitario di Scienze della Vita e Sanità Pubblica, Università Cattolica del Sacro Cuore, Rome, Italy; 4 Dipartimento Universitario di Scienze Radiologiche ed Ematologiche, Università Cattolica del Sacro Cuore, Rome, Italy

**Keywords:** Cervical cancer, Magnetic resonance imaging, Neoadjuvant chemotherapy, Fertility-sparing treatment, Conization

## Abstract

**Introduction:**

MRI is very accurate in selecting young women with cervical cancer for fertility-sparing surgery (FSS), in particular radical hysterectomy (RH). In order to improve obstetrical outcomes, neoadjuvant chemotherapy (NACT) followed by cold knife conization (CKC) has been proposed as alternative technique.

**Objective:**

To investigate the role of MRI in evaluation of response to treatment after neoadjuvant chemotherapy (NACT), followed by CKC, in patients with cervical cancer FIGO stage IB2-IIA1 with tumor size 2 – 4 cm, desiring to preserve their fertility.

**Methods:**

13 young women (23–36 years old) with cervical cancer stage IB2-IIA1 desiring to preserve their fertility were included. Tumor diameter at baseline and after treatment was detected on 1.5 T MRI. Treatment response was assessed according to Response Evaluation Criteria in Solid Tumors (RECIST 1.1) and then compared to histopathology result.

**Results:**

MRI correctly assessed 11 out of 13 cases, according to RECIST 1.1, compared to histopathology. Among these 7 patients with partial response (PR), 2 cases of CR, 1 SD and 1 PD with persistence or enlargement of primary tumor.

**Conclusion:**

Our pilot study supports the usefulness of MRI in assessment of treatment response after NACT, followed by CKC.

**Trial registration number:**

ClinicalTrials.gov: NCT02323841

## Introduction

Cervical cancer represents the fourth-most common cancer in women and the leading gynecologic malignancy, accounting approximately 570,000 cases and 311,000 deaths in 2018 worldwide [1]. In Europe, during last years, both mean age of pregnant women and number of nulliparous women have increased [2]. Age-specific incidence data indicate that 43% of patients with cervical cancer have less than 45 years [3]. As a consequence, numerous young women diagnosed with cervical cancer are still desiring to become pregnant.

In women with early stage cervical cancer (ECC), fertility-sparing surgery techniques include laparoscopic, abdominal or vaginal radical trachelectomy (RT) and cold knife conization (CKC) with or without pelvic lymph nodes dissection. Currently, RT is proposed as fertility-sparing surgery (FSS), alternative to radical hysterectomy, in patients with ECC staged IA1-IB1, as by the 2018 International Federation of Gynecology and Obstetrics (FIGO) classification [4].

According to a recent metanalysis, RT and CKC present similar oncologic outcomes in terms of recurrence rates, but CKC presents better obstetrical outcomes in terms of pregnancy rate (36% for CKC vs. 20% for RT) [5].

The National Cancer Comprehensive Network (NCCN) guidelines suggested RT as an alternative to radical hysterectomy (RH) in young women desiring to preserve fertility with [4, 6] disease limited to cervix, tumor size < 2 cm (or < 2.5 cm if exophytic lesion), absence of parametrial extension, an estimated distance of ≥ 1 cm from the proximal aspect of the tumor to the internal os, absence of lymphadenopathies or metastatic disease. Some authors have proposed neoadjuvant chemotherapy (NACT) followed by CKC as a safe and effective approach in ECC [7–9].

MRI is reported to be very accurate in selecting patients eligible for FSS [10, 11], in tumor size evaluation and deep stromal invasion assessment [12]. In the evaluation of parametrial invasion, the specificity and negative predictive value of MRI were 97% and 100%, respectively [13]. Furthermore, MRI has demonstrated very high sensitivity and specificity in assessment of internal os involvement, 90% and 98%, respectively. Moreover, MRI including diffusion-weighted imaging (DWI) assumes an important role in the evaluation of tumor response after chemotherapy [14, 15]. DWI with high resolution reduced field-of-view (FOV) improves tumor delineation [16]. In addition, MRI is highly sensitive and accurate (90.7% and 91.7%, respectively) in tumor size evaluation after FSS, especially in patients who have undergone cone biopsy before MRI examination [17]. 3 Tesla MRI is more reliable in assessment of parametrial invasion and uterine corpus invasion in early stage cervical cancer [18].

The purpose of this study was to investigate the role of MRI, including DWI, in the evaluation of response to treatment after NACT, followed by CKC, in patients with ECC (FIGO 2018 stage IB2-IIA1) with tumor size 2–4 cm, desiring to preserve their fertility.

## Methods and materials

### Study protocol

The present study is part of a single-center protocol prospectively aiming to assess oncological and the obstetrical outcomes of early stage IB2-IIA1 cervical cancer patients, as by the FIGO 2018 classification, with tumor size 2–4 cm, and wishing to preserve their fertility [19].

The study protocol consisting of neoadjuvant platinum/paclitaxel chemotherapy followed by conservative surgery (ClinicalTrials.gov: NCT02323841) was approved by the Institutional Ethical Committee (N. 12,650/13). All patients signed written informed consent in order to agree the procedures and for their data to be collected.

In the present study, we assess MRI performance, including DWI, in evaluating response to treatment after NACT followed by CKC. Histopathology was used as reference standard.

### Work-up and eligible criteria

Between March 2014 and the end of September 2018, 25 consecutive patients with cervical cancer FIGO stage IB2-IIA1 > 2 cm wishing to preserve fertility were prospectively enrolled.

Further inclusion criteria were histologically assessed diagnosis of cervical cancer, stage FIGO IB2-IIA1 with tumor diameter between 2 and 4 cm, squamous and adenocarcinoma histotype, absence of nodal disease, age 18—40 years, no menopausal status, wish to preserve fertility, refusal of radical treatment. Exclusion criteria were neuroendocrine cervical tumors or other rare histology, such as glassy cells or clear cell carcinoma, ongoing pregnancy at the time of diagnosis.

Preoperative and post-NACT assessment consisted of clinical examination, colposcopy, transvaginal pelvic ultrasound (US), abdominal-pelvic nuclear magnetic resonance imaging (MRI) and PET-CT. Tumor dimension was evaluated by MRI and physical examination. Lymph nodes status was determined by MRI preoperatively and then established at pathology after laparoscopic lymphadenectomy.

At the beginning of the study, all patients were submitted to pelvic lymphadenectomy. After the growing evidence about the role of sentinel lymph node biopsy (SLNB) in early stage cervical cancer (FIGO stage IA, IB1 and IB2) [20], starting from 2015, we considered this nodal assessment approach in our study, after adequate patient information and consent.

### NACT

After laparoscopic pelvic lymph node evaluation, eligible women underwent NACT administered for 3 cycles including cisplatin 70 mg/m^2^, and paclitaxel 175 mg/m^2^, every 21 days. After completion of NACT, the patients underwent clinical-instrumental re-assessment, including MRI. During chemotherapy, GnRH analogs or estroprogestin contraceptive pills in a continuous regimen, based on the patient choice, were used to protection of the ovarian reserve and to improve potential fertility.

### MRI protocol

All patients were scanned with a 1.5-T MR scanner using a standard 8-channel phased-array body coil. Patients are encouraged to fast 4 h and empty the bladder prior to MRI examination to reduce peristalsis-related motion artifacts. An anti-peristaltic agent is administered intramuscularly at the start of the examination to further reduce peristalsis. Conventional sequences included axial T1-weighted and T2-weighted sequences for panoramic pelvic evaluation and high-resolution T2-weighted sequences performed in the sagittal, axial oblique (perpendicular) and coronal oblique planes (parallel) along the long axis of the cervix. Diffusion-weighted imaging (DWI) is performed in the sagittal and axial oblique planes in the same orientation as T2-wieghted planes (b = 0 and 800–1000 s/mm^2^) and apparent diffusion coefficient (ADC) maps obtained. Large field-of-view single-shot fast spin echo (SSFSE) images from the top of the kidneys to the iliac crests are acquired to exclude hydronephrosis and para-aortic lymphadenopathy. Technical parameters are reported in Table 1.

**Table 1:** MRI acquisition parameters

Image sequences							
	Axial T1-W	Axial T2-W	Sagittal T2-W	Axial oblique T2-W (perpendicular to the long axis of the cervix)	Coronal oblique T2-W (parallel to the long axis of the cervix)	Axial oblique DW (= Axial oblique T2-W)	Axial abdominal T2-W
Sequence type	FSE	FRFSE	FRFSE	FRFSE	FRFSE	EPI	FRFSE
Echo time (ms)	16	85	85	85	85	Minimum	84
NEX	2	2	2	4	4	6	1
Repetition time (ms), TR	470	4500	4500	4500	4500	5425	1850
No. of sections	30	30	26	16	16	30	48
Receiver bandwidth (kHz)	31.25	31.25	41.67	41.67	41.67	–	41.67
Echo train length	3	26	15	26	26	–	–
Field-of-view (mm), FOV	240	240	240	220	240	280	280
Section thickness (mm)	4	4	4	3	4	4	4
Section spacing (mm)	0.5	0.5	0.4	0.5	0.5	0.5	0.5
Matrix size	448 × 288	384 × 256	384 × 256	384 × 256	384 × 256	128 × 128	128 × 128
b value (sec/mm^2^)	–	–	–	–	–	800	800
Phase direction	A/P	A/P	S/I	UNSWAP	UNSWAP	R/L	R/L

### Imaging analysis

Two radiologists, with multi-year expertise in gynecologic imaging, reviewed MRI images in consensus on a picture archiving and communication system (PACS) (Carestream Health, Rochester, NY). Firstly, T2-weighted images were evaluated. The readers had to detect the presence of intermediate or high T2-WI signal intensity of cervical carcinoma. Tumor diameters were assessed on sagittal, and oblique axial and coronal T2-WI. Maximum tumor diameter (TD) was chosen between the major diameter along the three axes. At baseline MRI examination, the readers evaluated eventual parametrial involvement, distance of the tumor from internal os and eventual vaginal extension of the tumor. Tumor diffusion restriction was qualitatively evaluated on DWI sequence. Last, occurrence of pelvic and/or lumboaortic lymphadenopathies was researched on conventional images.

After NACT conclusion, treatment response was assessed on MRI according to Response Evaluation Criteria in Solid Tumors (RECIST 1.1) [21]: complete response (CR): disappearance of the clinically evident disease, partial response (PR): reduction > 30% of the maximum tumor diameter; stable disease (SD): reduction of the tumor diameter not sufficient to define itself as PR; disease progression (PD): at least > 20% increase in the tumor diameter.

Radiological response to NACT was compared to histopathology result, representing our reference standard.

At follow-up MRI examination, tumor recurrence was investigated.

Surgical treatment(s) and assessment of pathologic response to NACT.

Patients with CR or PR were treated with CKC. Patients with SD or PD were proposed for radical hysterectomy (RH) and/or chemo-radiation. Pathological responses were defined as follows: complete disappearance of tumor in the cervix (pR0); remaining disease with ≤ 3 mm stromal invasion, including in situ carcinoma (pR1); residual disease with > 3 mm stromal invasion or multiple foci < 3 mm on surgical specimen (pR2) [22]. After CKC, in the presence of preneoplastic lesion (SIL) on the resection margins, the patient was counseled for re-conization by diathermic loop (LEEP/LLETZ) or for RH in the presence of residual neoplastic lesion on the resection margins. Histopathology result was considered the gold standard.

### Follow-up procedures

Clinical-instrumental follow-up was performed every 6 months in the first two years and every year from the third year on. Each follow-up visit includes gynecological examination, pap test, colposcopy and MRI. Patients could start their attempts to conceive only after the first six-months follow-up.

## Results

### Patients’ characteristics.

Between March 2014 and September 2018, 25 consecutive patients were enrolled.

8 out of 25 patients refused to participate, preferring RH as primary treatment option.

Four patients were not admitted after screening procedures: in 3 patients lymph node metastases were found (2 micro- and 1 macro-metastasis) and 1 patient, 40 years old, with AMH serum levels of 0.06 ng/ml, and FSH and LH levels underlying a pre-menopausal status, resulting in a final study population of 13 patients (Table 2).

**Table 2:** Patients’ characteristics at baseline

Patient ID	Age at diagnosis	Stage	T size mm	Histotype	Grade
#1	28	IB2	30	AD	2
#2	28	IB2	30	AD	2
#3	36	IB2	35	SCC	2
#4	36	IIA1	30	SCC	2
#5	26	IB2	30	SCC	3
#6	23	IB2	30	SCC	3
#7	23	IB2	25	SCC	3
#8	26	IIA1	30	AD	2
#9	35	IB2	40	SCC	3
#10	35	IB2	40	SCC	3
#11	30	IB2	30	AD	2
#12	34	IB2	25	AD	2
#13	29	IB2	22	AD	2

AD: adenocarcinoma; SCC: squamous cell carcinoma

All patients were of Caucasian race, 11 were Italian, 1 was born in Ukraine and 1 was born in Poland. The median age was 29 years (range 23–36 years). Seven patients (53.8%) had squamous cell carcinoma (SCC), and 6 (46.1%) adenocarcinoma (AD). Grade of tumor differentiation was G2 in 8 patients (61.5%), and G3 in the remaining 5 (38.5%).

The mean time between the end of NACT and MRI imaging was 19 days (range: 8–55).

At the end of March 2020, the median follow-up was of 37 months (range: 18–76). In this group, 1 distant recurrence in the liver, in the absence of loco-regional recurrence, was detected at 1^st^ follow-up MRI; patient was managed by atypical liver resection and RH and still alive without evidence of disease after 72 months.

Before data collection, only 3 patients tried to conceive and 2 became pregnant, both spontaneously. The 2 patients underwent a cesarean section (at 34 weeks because of pPROM and at 37 weeks, because of PROM, respectively). The newborns are currently in good conditions.

### Tumor response evaluation.

The median maximum tumor diameter assessed at baseline MRI was 30 mm (range 22–40 mm). Median maximum tumor diameter assessed at post-treatment MRI was 9 mm (range 0–40 mm). Three patients achieved CR (23.1%), and 8 patients (61.5%) showed PR. The two patients remaining experienced SD (15,3%) and were treated with RH; one of them experienced PD (7.7%) and died of disease after 15 months (Table 3).

**Table 3:** Response evaluation according to RECIST 1.1 and pathologic response

Patient N°	Tumor response (according to RECIST 1.1)	pR (pathologic response)
#1	PR	pR1 (1 mm)
#2	PR	pR2 (length 10 mm, invasion 6 mm)
#3	PR	pR1 (1.5 mm)
#4	PR	pR1 (< 0.5 mm)
#5	CR	pR0
#6	PD	
#7	PR	pR1 (length 4 mm, invasion 3 mm)
#8	PR	pR1 (< 2 mm)
#9	SD	
#10	CR	pR1 (< 0.5 mm)
#11	PR	pR2 (18 mm)
#12	CR	pR0
#13	PR	pR0

pR0: no detectable tumor in the surgical specimen; pR1: microscopic disease with ≤ 3 mm stromal invasion or in situ carcinoma; pR2: macroscopic residual disease with > 3 mm stromal invasion

### Histological evaluation.

Eleven responding patients were considered eligible for FSS and all underwent CKC. At conclusive analysis of surgical specimens, the absence of residual disease (pR0) was observed in 3 patients (27.3%). The presence of residual disease was observed in 8 cases, respectively, with macroscopic disease (pR2: > 3 mm stromal invasion) in 2 cases (18.2%) and microscopic disease (pR1: ≤ 3 mm stromal invasion) in 6 cases (54.5%) (Table 3).

### MRI performance in tumor response assessment.

A case was considered correctly classified when imaging assessment according to RECIST 1.1 (CR, PR, SD or PD) corresponded to histopathological result (pR0, pR1, pR2) (Table 3).

**Fig. 1:**
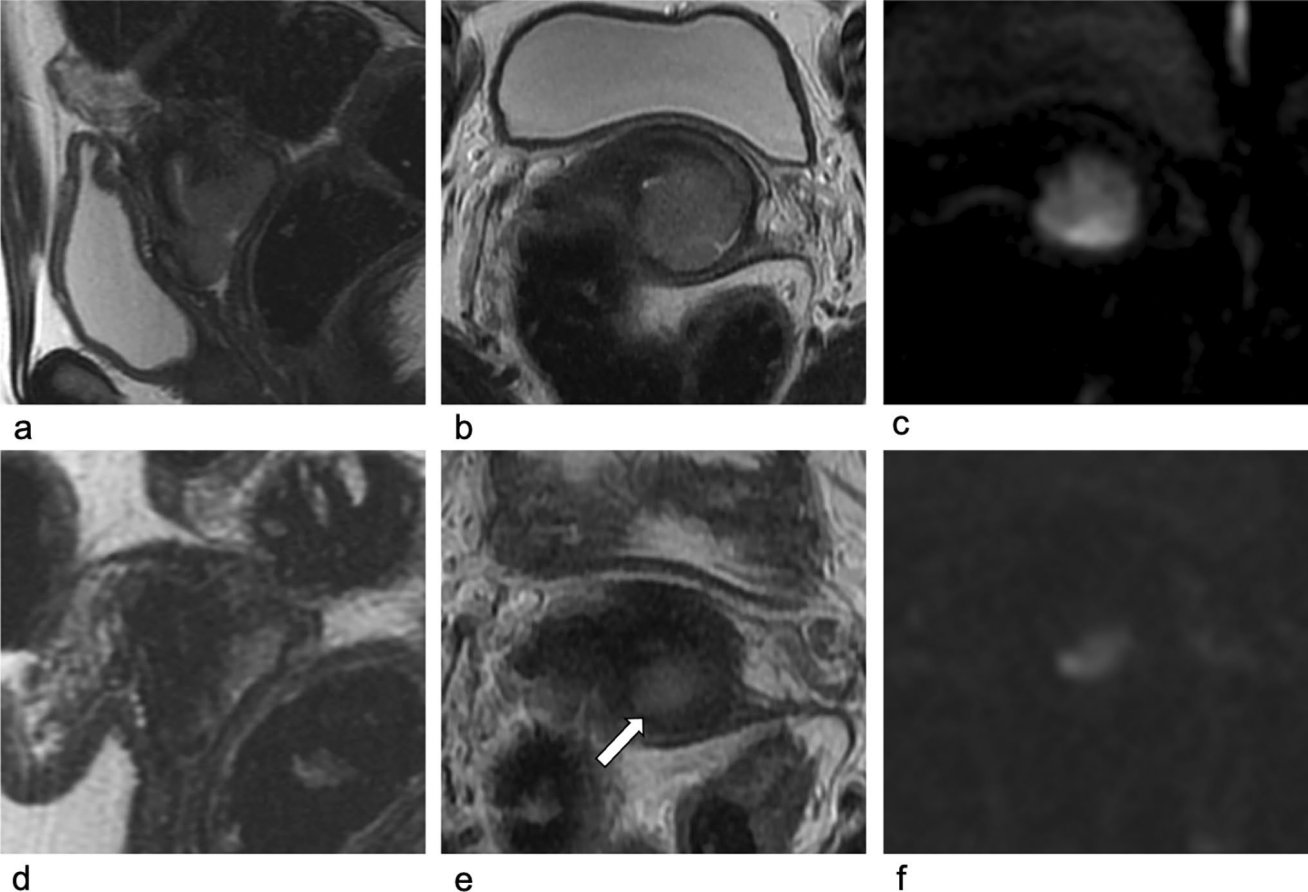
Baseline and post-NACT MRI in 26-year-old woman with cervical adenocarcinoma (FIGO IB2). **a, d** Sagittal T2-w images. **b**, **e** Axial oblique T2-weighted images. **c**, **f** Axial oblique DWI images (b value 800 s/ mm^2^). At baseline MRI (**a**, **b**, **c**), T2-w sequences show the hyperintense cervical mass, extending to the upper third of the vagina, with maximum diameter of 30 mm (any plane). The lesion shows high signal intensity also on DWI sequence (**c**). After NACT (**d**, **e**, **f**), T2-w sequences show the presence of an area of hyperintensity in the cervix (arrow in **e**), to which corresponds to a high-signal-intensity area also on DWI images (**f**), configuring a PR. NACT: neoadjuvant chemotherapy; PR: partial response

MRI correctly assessed 11 out of 13 cases: 7 patients with PR (Fig. 1) corresponding to 2 patients with macroscopic residual (pR2) and 5 with microscopic residual (pR1), 2 cases of CR, assessed as PR0 on histology, 1 SD and 1 PD with persistence or enlargement of primary tumor.

One patient identified as PR on MRI had no residual disease (pR0) on surgical specimen. One patient classified as CR had still evidence of disease on histology, presenting microscopic residual (pR1).

## Discussion

**Fig. 2:**
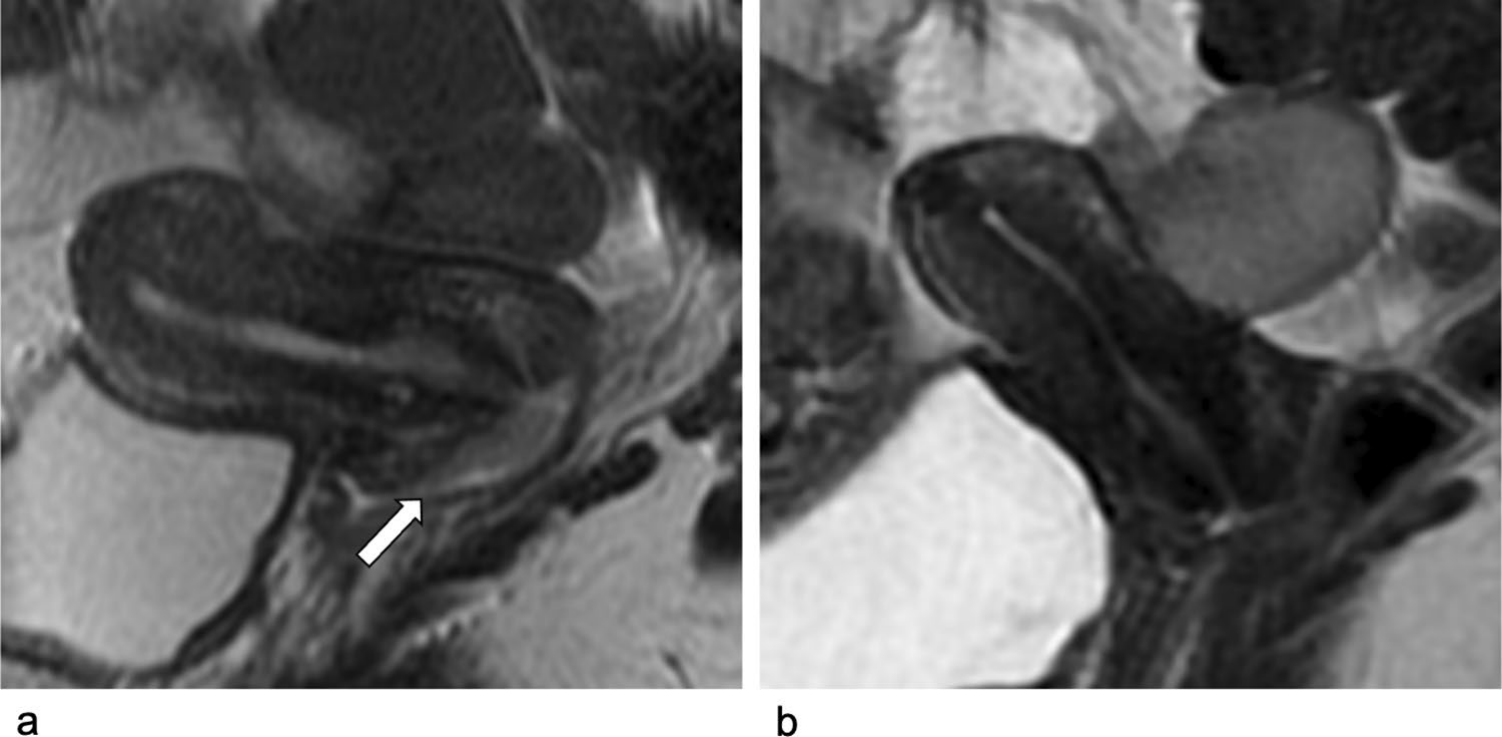
Baseline and post-NACT MRI in 34-year-old woman with cervical adenocarcinoma (FIGO IB2). **a**, **b** Sagittal T2-w images. At baseline MRI (**a**), sagittal T2-w images show the hyperintense cervical mass (arrow), with maximum diameter of 25 mm. After NACT (**b**), T2-w sequences show no evidense of residual disease both on T2-w images (**b**, **c**) and on DWI images (**d**), configuring a CR to NACT. NACT: neoadjuvant chemotherapy; CR: complete response

The results from our series confirm the usefulness of MRI, including DWI, in tumor response evaluation after NACT in patients with ECC (FIGO 2018 stage IB2-IIA1). In fact, tumor response was properly assessed in 11 out of 13 cases, investigating both conventional and diffusion-weighted images. In 1 out of 13 patients, CR was assigned at post-NACT evaluation (Fig. 2); on histology, this patient had a microscopic residual ≤ 3 mm (pR1).

MRI has a primary role in cervical cancer staging [1], fertility-sparing surgery selection [10] and monitoring treatment response after NACT or chemoradiotherapy in locally advanced cervical cancer (LACC) [15, 23]. Several post-chemotherapy modifications, such as edema, inflammation and necrosis can mimic tumor residual on conventional imaging [24]. The addition of DWI increased MRI performance in tumor response assessment after non-surgical treatment [23]. In our series, only 1 out of 13 case was configured as PR on MRI, while the absence of residual disease (pR0) was detected on histological sample. In this patient, post-NACT MRI was performed only 8 days after the end of treatment. The short interval time between the end of treatment and MRI examination could be cause of false positive MRI interpretation as already reported in the literature in the evaluation of tumor response after chemo-radiation therapy or radiotherapy. In fact, the optimal time between neoadjuvant therapy (chemo-radiation therapy or radiotherapy alone) and post-treatment MRI has been reported to be 4–6 weeks or 3 months, in patients with locally advanced cervical cancer [14, 25]. However, there are no currently studies investigating MRI timing in response evaluation after chemotherapy alone in cervical cancer.

To our knowledge, there are no studies currently in literature investigating MRI performance in tumor response assessment in ECC treated with NACT followed by CKC. MRI was used to assess clinical response after NACT only in the retrospective study published by Salihi et al. [8], including 11 patients, but its performance was not investigated. Some differences in study population are present between our series and Salihi’s. Only patients with maximum tumor diameter between 2 and 4 cm were prospectively enrolled in our study, while Salihi et al. [8] included patients with tumor size ≤ 3 cm, with a median tumor diameter of 15 mm. Tumor size was < 2 cm in 7 patients (64%) and > 2 cm and in 4 cases (36%) [8]. Other authors assessed the effectiveness of NACT followed by CKC [7] or trachelectomy [26], but MRI performance was not investigated.

**Fig. 3:**
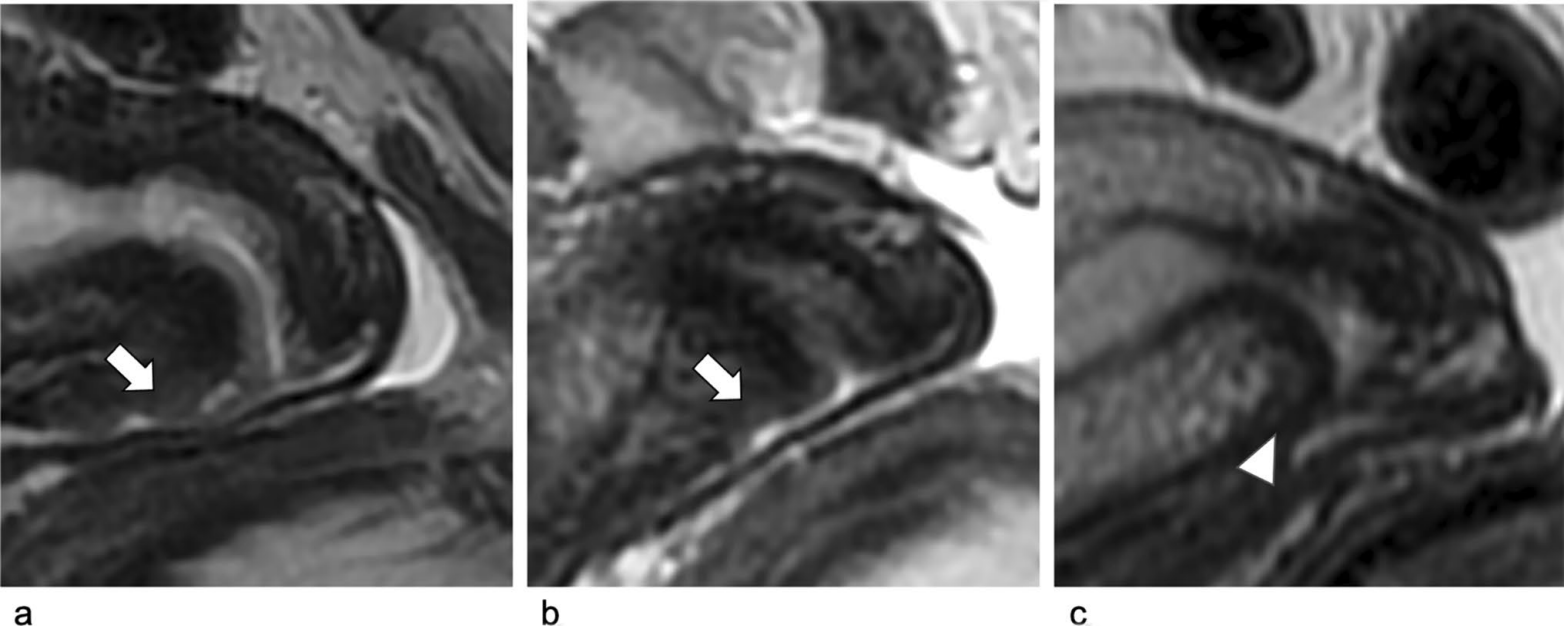
Post-conization MRI in a 29-year-old woman with cervical adenocarcinoma (FIGO staging IIA1), who underwent CKC after NACT. Sagittal T2-w images at baseline (**a**), after NACT (**b**) and after CKC (**c**). Baseline MRI shows the hyperintense cervical mass (arrow in **a**). Post-NACT shows reduction of the cervical lesion with persistence of a small hyperintense area within the anterior cervical lip (arrow in **b**), with diffusion restriction on DWI and ADC map (not shown). Post-conization sagittal T2-w images (**c**) show modfications represented by shortening of the cervical canal, and reduction of dimension of both anterior and posterior lips, better evident on the anterior lip (arrowhead in **c**.)

**Fig. 4:**
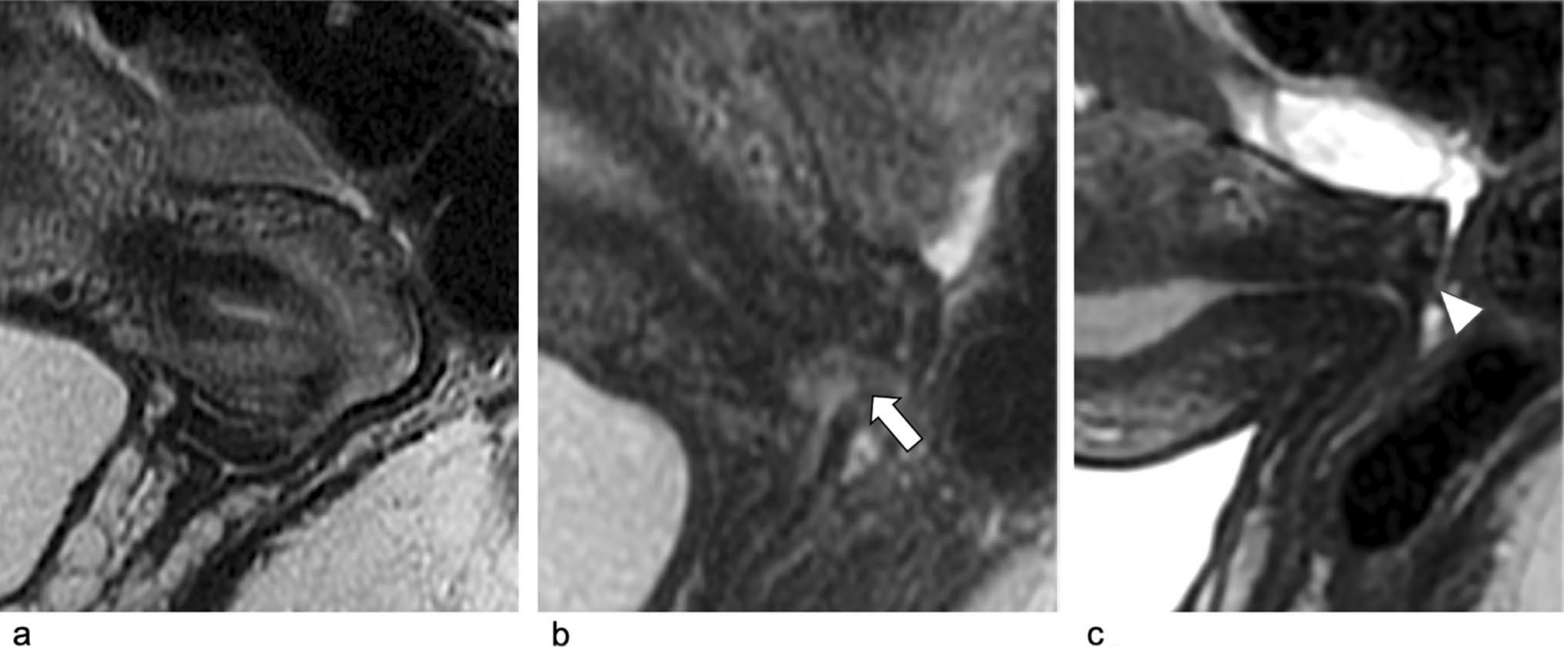
Post-conization MRI in a 36-year-old woman with cervical squamous cell carcinoma (FIGO IIA1), who underwent CKC after NACT. Sagittal T2-w images at baseline (**a**), after NACT (**b**) and after CKC (**c**). Baseline MRI shows the hyperintense cervical mass (**a**). Post-NACT shows reduction of the cervical lesion with persistence of a small hyperintense area within the posterior cervical lip (arrow in **b**), with diffusion restriction on DWI and ADC map (not shown). Post-conization sagittal T2-w images (**c**) show significant shortening of both anterior and posterior lips (arrowhead in **c**)

After CKC, cervix appearance on MRI may vary according to the depth of the conization. High signal intensity in the cervical region may be depicted on T2-weighted images, causing it to look smaller than usual [27] (Figs. 3–4). As resultant, asymmetry in the length of the anterior or posterior lips of the cervix should be also considered during imaging follow-up, in order to avoid residual misdiagnosis [10]. In our series, cervical shape modifications were observed and correctly diagnosed as post-conization modifications, being aware of CKC location. Ideal cervical length, measured on sagittal T2WI from the internal to external os, should be > 2.5 cm [11]. In case of extra conization, this information is important to avoid obstetrical complications, such as cervical incompetence and future preterm delivery.

Moreover, DWI has demonstrated to be helpful in differentiate between post-treatment modifications and residual disease after CKC [28]. According to this paper, DWI was helpful, in doubtful cases, in distinguishing residual tumor from other different aspects related to previous chemo- and surgery treatments in our experience.

The advantages of the present study are represented by prospective construction, homogeneity of series, including patients with same tumor size, treatment workflow, complete imaging dataset and histopathological analysis as gold standard.

The present study has some limitations. First, the choice to cluster patients with microscopic and macroscopic residual disease on histopathology in the PR group. This decision was supported by data on prognosis of patients with microscopic residual disease (pR1), which is worse than that of patients with the absence of residual disease (pR0) [29]. Other limits are small sample size and long-recruitment time (2014–2018), which can be explained by the proposal of an experimental approach (NACT followed by CKC) in a restricted population (FIGO staging IB2-IIA1 with tumor size 2–4 cm, desiring to preserve their fertility). The variable lead time between the end of NACT and MRI represents another limit of the study, due to small sample size; in fact, only one patient underwent MRI 55 days after the end of NACT.

In conclusion, considering increasing incidence of cervical cancer among young women, several fertility-sparing treatment options have been proposed, in order to obtain successful oncological and obstetrical outcomes. As consequence, imaging assumes a crucial role in tumor response evaluation and follow-up of these patients. In this context, our results encourage the use of MRI in assessment of tumor response in patients with ECC desiring to preserve their fertility treated with NACT followed by CKC, even if larger studies are necessary to validate our data.

## Data Availability

All patients signed written informed consent in order to agree the procedures and for their data to be collected.

## Abbreviations

CKC: Cold knife conization
CR: Complete response
DWI: Diffusion-weighted imaging
ECC: Early stage cervical cancer
FSS: Fertility-sparing surgery
LACC: Locally advanced cervical cancer
NACT: Neoadjuvant chemotherapy
PACS: Picture archiving and communication system
PD: Disease progression
PR: Partial response
RH: Radical hysterectomy
RT: Radical trachelectomy
SD: Stable disease
TD: Tumor diameter
